# Beyond the Alarmin: Nuclear Interleukin-33 and Skin Barrier Dysfunction in Atopic Dermatitis

**DOI:** 10.3390/jcm15207781

**Published:** 2026-10-09

**Authors:** Jun Muto

**Affiliations:** Department of Dermatology, Ehime University Graduate School of Medicine, Ehime 791-0295, Japan; junmuto@m.ehime-u.ac.jp

**Keywords:** nuclear IL-33, skin barrier dysfunction, atopic dermatitis, filaggrin, STAT3, alarmin, keratinocyte, trehalose

## Abstract

Interleukin-33 (IL-33) belongs to the IL-1 superfamily and is defined by functional duality; it resides constitutively within barrier epithelial cell nuclei under homeostatic conditions but is released into the extracellular compartment, where it operates as an alarmin that initiates ST2-dependent type 2 inflammation, upon cellular damage. In atopic dermatitis (AD), IL-33 is markedly overexpressed in lesional keratinocytes, and its extracellular cytokine activity has long been regarded as the principal disease-relevant function. However, accumulating evidence indicates that nuclear IL-33 itself may play an active, receptor-independent role in keratinocyte biology. This review reconciled two apparently opposing paradigms of nuclear IL-33 whereby it acts as (1) a passive sequestration checkpoint that restrains alarmin activity in several cell types and (2) an active transcriptional regulator of the epidermal barrier in keratinocytes. We analyzed the proposed role of nuclear IL-33 as a putative intracellular hub of barrier dysfunction by summarizing how the major type 2 cytokines in AD (i.e., IL-4/IL-13, IL-31, and TSLP) converge on it to suppress barrier and differentiation genes, as well as filaggrin, loricrin, keratins, claudin-1, and antimicrobial peptide. We further explored the limited clinical efficacy of extracellular IL-33/ST2-targeting biologics in AD, compared with that of nuclear-IL-33-directed strategies, including topical trehalose, which are barrier-restorative, mechanistically targeted therapeutics. As this model derives largely from in vitro keratinocyte and three-dimensional skin studies, nuclear IL-33 should therefore be regarded as a putative, context-dependent cofactor whose direct chromatin action and in vivo relevance remain to be established.

## 1. Introduction

Interleukin-33 (IL-33) belongs to IL-1 superfamily and is a tissue-derived nuclear cytokine that is abundantly expressed in endothelial cells, epithelial cells, and fibroblast-like cells during homeostasis and inflammation. Upon necrotic cell death resulting from tissue injury, IL-33 is released into the extracellular space, where it functions as a damage-associated molecular pattern or alarmin to initiate and amplify innate immune responses through its receptor ST2 (also known as IL-1 receptor-like 1, IL1RL1) [1,2,3,4]. In atopic dermatitis (AD), IL-33 was found to be overexpressed in lesional keratinocytes [5]; moreover, transgenic mice expressing IL-33 under the control of a keratin 14 promoter spontaneously developed AD-like dermatitis, providing direct evidence for a causal role of keratinocyte-derived IL-33 in AD pathogenesis [6]. Since its discovery, IL-33 has been regarded as a central orchestrator of type 2 immunity at barrier surfaces, where it serves as an early sentinel that alerts the immune system to epithelial damage.

The N-terminal portion of IL-33 contains a chromatin-binding motif (CBM) and a predicted bipartite nuclear localization sequence (NLS). Accordingly, endogenous IL-33 is constitutively expressed at high levels in the nuclei of producing cells, where it associates with chromatin [4,7,8]. Despite this conspicuous nuclear localization, the pathogenic role of nuclear IL-33 in allergic diseases has remained poorly defined, and research has historically focused on the released extracellular form.

We have previously shown that epidermal barrier impairment in AD involves regulation of type 2 cytokine-driven skin barrier dysfunction by nuclear IL-33 in keratinocytes and have developed a therapeutic strategy that targets the nuclear function of IL-33 [9,10,11,12]. In this review, we summarized the clinical relevance of IL-33 in AD and discussed the molecular mechanisms through which nuclear IL-33 regulates skin barrier homeostasis. Importantly, we placed these findings within a broader unresolved debate on whether nuclear IL-33 functions principally as a passive sequestration checkpoint that restrains alarmin activity or as an active context-dependent transcriptional regulator. We propose that in the inflamed epidermis, nuclear IL-33 may be repurposed as an intracellular signaling hub that links type 2 cytokine signals to barrier gene repression and considered the therapeutic implications of this perspective. By integrating structural, mechanistic, and clinical evidence, this review aimed to provide a unified framework for understanding nuclear IL-33 as a regulator of the epidermal barrier and to delineate the resulting translational opportunities.

## 2. IL-33 Biology: Structure, Receptor, Signaling, and Cellular Sources

IL-33 is organized into three functional regions: an N-terminal nuclear domain harboring CBM and NLS, a central protease-sensitive region, and a C-terminal IL-1-like cytokine domain that engages the receptor [2,4]. The extracellular cytokine binds membrane-bound ST2 and recruits the coreceptor IL-1 receptor accessory protein (IL-1RAcP); the resulting ST2/IL-1RAcP complex signals through MyD88, IRAK1, IRAK4, and TRAF6 to activate the NF-κB and MAPK cascades [1,2,3,4]. A soluble form of ST2 (sST2) acts as a decoy receptor that buffers extracellular IL-33 activity [3].

The principal target cells of IL-33 are tissue-resident, ST2-expressing immune cells, including mast cells, basophils, dendritic cells, eosinophils, group 2 innate lymphoid cells (ILC2s), tissue regulatory T cells, T helper (Th) 1 and 2 cells, NK cells, B cells, and macrophages; structural cells, such as epithelial cells, fibroblasts, endothelial cells, and neural cells can also respond [3]. When released after injury, IL-33 acts on these ST2-bearing cells to induce type 2 responses, which are characterized by IL-4, IL-5, and IL-13 production [1].

Under homeostatic conditions, IL-33 is constitutively expressed in the nuclei of endothelial cells; fibroblast-like cells; and, notably, epithelial cells that line barrier tissues, such as the skin, gut, and lungs, as well as lymphoid fibroblastic reticular cells and glial cells of the nervous system [4]. IL-33 expression is further enhanced during inflammation, such as in keratinocytes and dermal endothelial cells of patients with AD [5]. In the lungs, alveolar type II epithelial IL-33 expression was found to increase after nematode infection; cigarette smoke exposure; and intranasal challenge with allergens, such as ragweed pollen or Alternaria [13,14]. Moreover, nasal epithelial IL-33 is essential for the development of allergic rhinitis in mice [15]. These observations established barrier epithelia as a dominant and inducible source of IL-33. Collectively, these properties position IL-33 as a sentinel cytokine, which couples epithelial barrier structural integrity with the activated state of the local immune compartment.

## 3. Molecular Basis of Nuclear IL-33

### 3.1. Nuclear Targeting and Chromatin Binding

Endogenous IL-33 is targeted to the nucleus by its N-terminal nuclear domain. The bipartite NLS mediates nuclear import, whereas a short CBM docks into the acidic pocket formed by the histone 2A–2B dimer to tether IL-33 to the nucleosome surface. This mode of chromatin attachment is strikingly similar to that used by the Kaposi sarcoma herpesvirus LANA protein, an example of molecular mimicry between a host cytokine and a viral chromatin-anchoring factor [7,8]. Therefore, the majority of cellular IL-33 resides on chromatin rather than in a readily secreted pool. For clarity, we distinguish IL-33 species by processing state and by subcellular location. Full-length IL-33 denotes the intact, uncleaved protein, which is itself bioactive; within intact cells it resides predominantly as nuclear (chromatin-bound) IL-33, sequestered on chromatin, whereas upon cell damage it is released as extracellular full-length IL-33. Proteolytically processed (mature) IL-33 (see Section 3.3) refers to the shorter, more potent forms generated from the full-length protein by inflammatory-cell proteases after release.

### 3.2. Nuclear Sequestration as a Regulatory Checkpoint

A central concept emerging from in vivo studies is that nuclear localization is not merely for storage but is a checkpoint that restrains the potent alarmin activity of IL-33. In a knock-in mouse model, disruption of the N-terminal nuclear domain, which abolishes nuclear retention, was found to lead to constitutive extracellular leakage of IL-33; even heterozygous animals developed severe, nonresolving multiorgan inflammation with dense infiltrates of eosinophils, monocytes, neutrophils, and macrophages and died within roughly 3 months of birth [16]. Consistently, chromatin binding governs the kinetics of IL-33 release: compared with chromatin-binding-deficient truncated forms, wild-type IL-33 is markedly less mobile within the nucleus and far more resistant to necrosis-induced release; this way, nuclear tethering effectively buffers the amount of bioactive alarmin that reaches the extracellular space [17]. In this framework, the nucleus acts as a reservoir that prevents inadvertent or excessive IL-33 signaling. The clear physiological rationale of this buffering function is the unrestrained release of an already bioactive full-length IL-33, which would risk indiscriminate immune activation; therefore, chromatin tethering provides a spatial safeguard, which scales alarmin availability to the severity of tissue injury.

### 3.3. Proteolytic and Oxidative Regulation of Extracellular Activity

The activity of released IL-33 is further refined by post-release processing. Full-length IL-33 is biologically active, but inflammatory cell proteases, including neutrophil elastase and cathepsin G, and mast cell-derived chymase and tryptase, cleave the central region to generate mature forms with substantially enhanced potency [18]. Conversely, silent apoptotic death does not trigger inflammation, because apoptotic caspases (i.e., caspase-3 and caspase-7) cleave and inactivate IL-33, whereas necrotic death releases the active alarmin [19]. Once extracellular, IL-33 is rapidly oxidized, and the conformational change brought about by disulfide bridge formation abolishes ST2 binding, thereby limiting the spatial and temporal range of IL-33 signaling [20]. Together with nuclear sequestration, these layers of regulation underscore the normally tight control of the alarmin function of IL-33. These converging controls illustrate a broader principle that the restrained proinflammatory potential of IL-33 is through a hierarchy of concerted nuclear, proteolytic, and redox checkpoints rather than via a single mechanism.

### 3.4. Does Nuclear IL-33 Regulate Transcription? An Unresolved Question

The issue of nuclear IL-33 having an intrinsic receptor-independent function beyond sequestration remains debated and has conflicting data in the literature. In primary human endothelial cells, silencing of endogenous nuclear IL-33 did not result in reproducible proteome changes, whereas exogenous extracellular IL-33 induced a clear inflammatory protein signature [21]. Moreover, IL-33 overexpression did not alter global gene expression in transduced epithelial cells [17]. Consistently, elimination of IL-33-driven transcriptional response in the intestinal epithelium after genetic ablation of ST2 was attributed to the mature cytokine rather than to nuclear IL-33 [22]. These data argued that, at least in some cell types and in the resting state, the nuclear pool is functionally inert and mainly acts to restrain alarmin. In other settings, however, nuclear IL-33 appeared to act intracellularly; it was found to inhibit early conversion to an extracellular matrix-secreting phenotype in a receptor-independent manner in fibroblasts [23], modulate chromatin, and repress NF-κB-dependent transcription [24]. As detailed below, our work on keratinocytes added another disease-relevant dimension to this debate by showing that under a type 2 cytokine milieu, nuclear IL-33 becomes an active repressor of epidermal barrier genes [12]. Such divergent observations caution against extrapolating from one cell type to another and, instead, favor a context-specific framework, in which the functional output of the nuclear pool is dictated by the surrounding signaling environment.

## 4. Atopic Dermatitis and the Skin Barrier

AD is a chronic, relapsing inflammatory skin disease characterized by intense pruritus and mutual reinforcements of epidermal barrier dysfunction and type 2 inflammation [25]. In healthy skin, stratum corneum proteins, such as filaggrin (FLG); tight junction proteins, such as claudin-1 (CLDN1); and intercellular lipids act together to restrict transepidermal water loss (TEWL) and prevent allergen and microbe penetration. In AD, this barrier is compromised by reduced barrier protein expression and tight junction and lipid abnormalities, thereby increasing TEWL and facilitating entry of exogenous antigens and microorganisms. At the genetic level, loss-of-function mutations in the gene encoding FLG constitute the strongest known predisposing factor for AD and are associated with early-onset, persistent, and more severe disease. In addition, FLG degradation products contribute to stratum corneum hydration and maintenance of an acidic surface pH, which constrains microbial colonization. However, even in patients without *FLG* mutations, type 2 cytokines can impose a functionally FLG-deficient state by transcriptionally repressing barrier genes, resulting in convergence of inflammation and genetics to a common barrier-defective endpoint. Superimposed dysbiosis, such as abundance of *Staphylococcus aureus*, further amplifies barrier damage and type 2 inflammation, reinforcing the self-sustaining characteristic of the disease.

The resulting type 2-skewed immune response, which is dominated by Th2 cells and innate effectors, drives the expression of IL-4, IL-13, IL-31, and TSLP, among other mediators, to promote chronic inflammation and aggravate pruritus and scratching. Mechanical scratching and a type 2 cytokine environment further impair the barrier and promote alarmin release, sustaining AD in a self-amplifying itch–scratch cycle, in which barrier impairment and type 2 inflammation are inseparable [25,26]. IL-33, which is overexpressed in AD keratinocytes [5], is positioned as a critical mediator that links these two processes. The clinical corollary is that barrier repair and anti-inflammatory therapy are interdependent rather than independent objectives, because durable disease control requires interruption of reciprocal reinforcement between barrier failure and type 2 inflammation.

## 5. IL-33 in Atopic Dermatitis: A Dual Pathogenic Role

### 5.1. Extracellular IL-33 as a Driver of Type 2 Inflammation

IL-33 expression is markedly increased in AD keratinocytes, with particularly strong nuclear staining in the lesional epidermis [12]. Keratinocyte-specific IL-33 overexpression is sufficient to elicit AD-like dermatitis in mice [6]. Mechanistically, IL-33 stimulates ILC2s to secrete IL-5 and IL-13, and IL-4 released by IL-33-activated basophils further amplifies ILC2 responses [6,26]. Moreover, IL-33 induces the key pruritogenic cytokine IL-31, and the act of scratching itself promotes IL-33 release from keratinocytes, making IL-33 a shared molecular origin of the itch–scratch cycle [26]. Considering these, extracellular IL-33 operates at the apex of type 2 inflammatory cascade, translating epithelial injury into coordinated mobilization of ILC2s, Th2 cells, mast cells, basophils, and eosinophils, which drive type 2 immunity.

### 5.2. Extracellular IL-33 as a Direct Barrier-Disrupting Factor

Beyond its inflammatory effects, extracellular IL-33 directly weakens the epidermal barrier. In cultured human keratinocytes and three-dimensional skin models, exogenous IL-33 was reported to significantly downregulate FLG [27]. Furthermore, IL-33 was found to reduce FLG by inducing phosphorylation of ERK and STAT3 [28] and suppress CLDN1 through the ERK/STAT3 pathway, with activated STAT3 binding the CLDN1 promoter to directly inhibit transcription [29]. Therefore, even before considering intracellular functions, IL-33 is doubly pathogenic in AD, acting as an inflammatory inducer and a barrier-disrupting factor. Notably, these ST2-mediated extracellular effects converge on the same STAT3-dependent mechanism underlying the nuclear IL-33 pathway, which is detailed in Section 6, suggesting that extracellular and nuclear IL-33 engage a common transcriptional program.

The barrier defect in AD also extends to the epidermal lipid matrix. Type 2 cytokines suppress the fatty-acid elongases ELOVL3 and ELOVL6 in keratinocytes in a STAT6-dependent manner, shifting the ceramide profile toward shorter-chain species, reducing very-long-chain fatty acids, and thereby increasing transepidermal water loss [30]. Because this lipid remodeling is driven by the same STAT6 axis engaged by nuclear IL-33, IL-33-initiated type 2 signaling is well positioned to contribute to lipid-barrier impairment, although a direct link between IL-33 and elongase regulation has not yet been demonstrated and warrants dedicated study.

### 5.3. IL-33 and the Cutaneous Neuroimmune Axis

Beyond its actions on immune and structural cells, IL-33 also participates in the neuroimmune circuitry underlying pruritus in AD. Its receptor ST2 is expressed on a subset of sensory neurons, and keratinocyte-derived IL-33 can act directly on them to evoke itch, reinforcing the itch–scratch cycle together with IL-31 [31,32]. IL-33 is likewise linked to neuropeptide-driven inflammation: it augments substance P-induced mediator release from mast cells, while epidermal PAR2-TRPV3 signaling that drives keratinocyte IL-33 may in turn regulate substance P expression and sensory nerve–mast cell interactions [33,34]. Whether the nuclear IL-33 pool contributes to these largely ST2-dependent interactions is presently unknown and warrants investigation; nonetheless, integrating them suggests that a single alarmin may couple barrier dysfunction, type 2 inflammation, and pruritus within one network [35].

## 6. Nuclear IL-33 as an Intracellular Hub of Type 2 Cytokine-Induced Barrier Dysfunction

Given the abundant nuclear IL-33 observed in lesional keratinocytes, we investigated whether this nuclear pool serves a function beyond being a releasable alarmin. Based on a series of studies, the major type 2 cytokines, which are increased in AD, converge on nuclear IL-33 to impair keratinocyte differentiation and barrier integrity, supporting a role for nuclear IL-33 as a putative intracellular signaling node downstream of multiple receptors [10,11,12].

### 6.1. IL-4/IL-13

**Figure 1:**
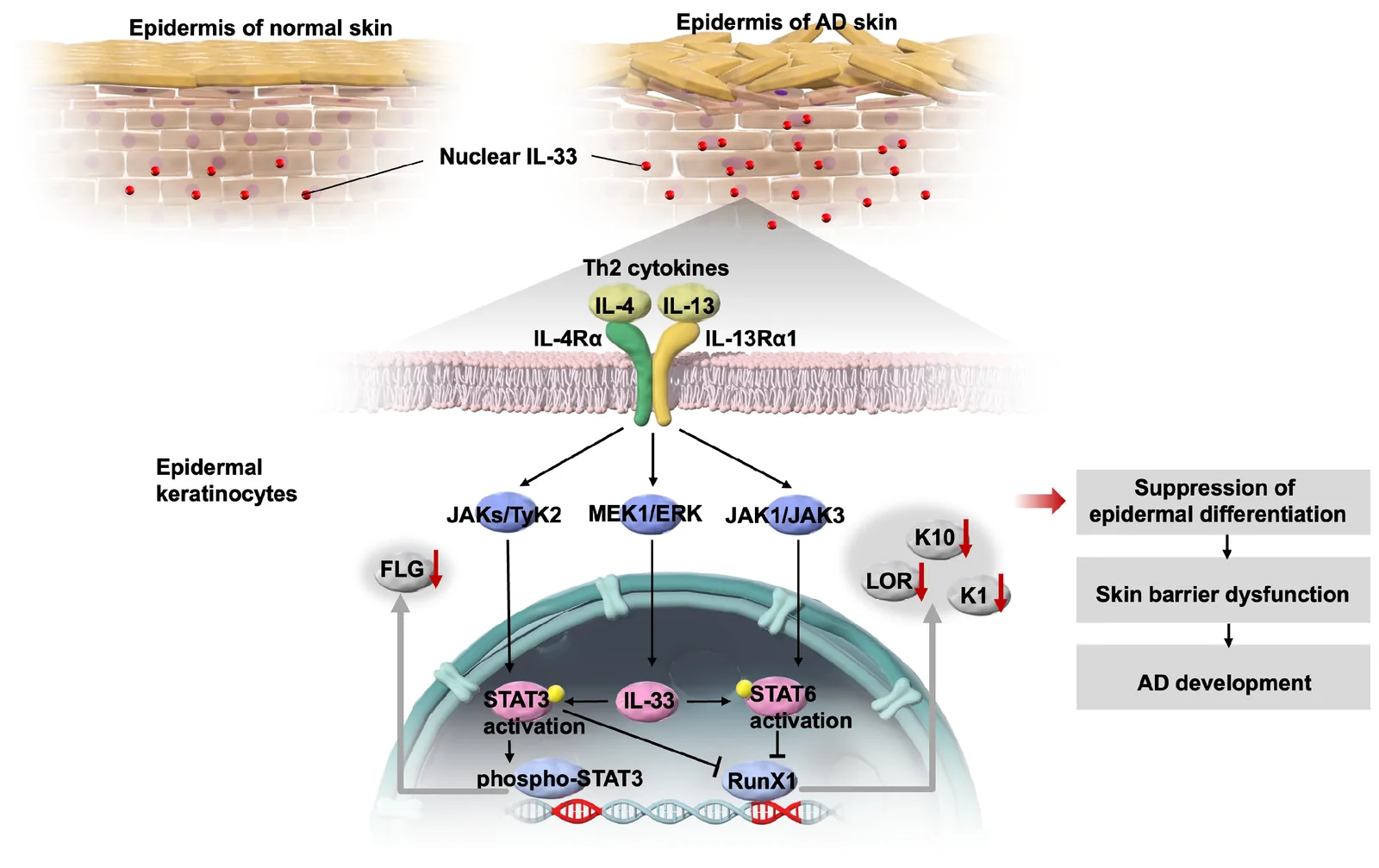
Central role of nuclear IL-33 in IL-4/IL-13-induced suppression of filaggrin, loricrin, and keratin 1/10 in keratinocytes. Adapted with permission from Ref. [12]. Copyright [2021], Elsevier.

Stimulation of human keratinocytes with IL-4/IL-13 increased the nuclear abundance of full-length IL-33, an effect that required activation of the MEK/ERK pathway. Preventing nuclear IL-33 increase attenuated IL-4/IL-13-induced suppression of the differentiation and barrier genes *FLG*, *loricrin* (*LOR*), and *keratins 1* and *10* (*KRT1/KRT10*) and restored barrier function. Mechanistically, nuclear IL-33 enhanced the binding of phosphorylated STAT3 (pSTAT3) to the FLG promoter, thereby repressing FLG transcription; in parallel, STAT3 and STAT6 suppressed the transcription factor RUNX1, leading to decreased LOR and KRT1/KRT10 [12]. These findings support the nuclear IL-33/STAT3/STAT6 axis as a contributor to IL-4/IL-13-induced barrier impairment (Figure 1). The dependence of nuclear IL-33 accumulation on MEK/ERK signaling indicates that barrier gene suppression is an actively controlled, signal-driven process rather than a passive response to cytokine exposure, thereby defining a discrete and pharmacologically tractable node within the pathway.

### 6.2. IL-31

**Figure 2:**
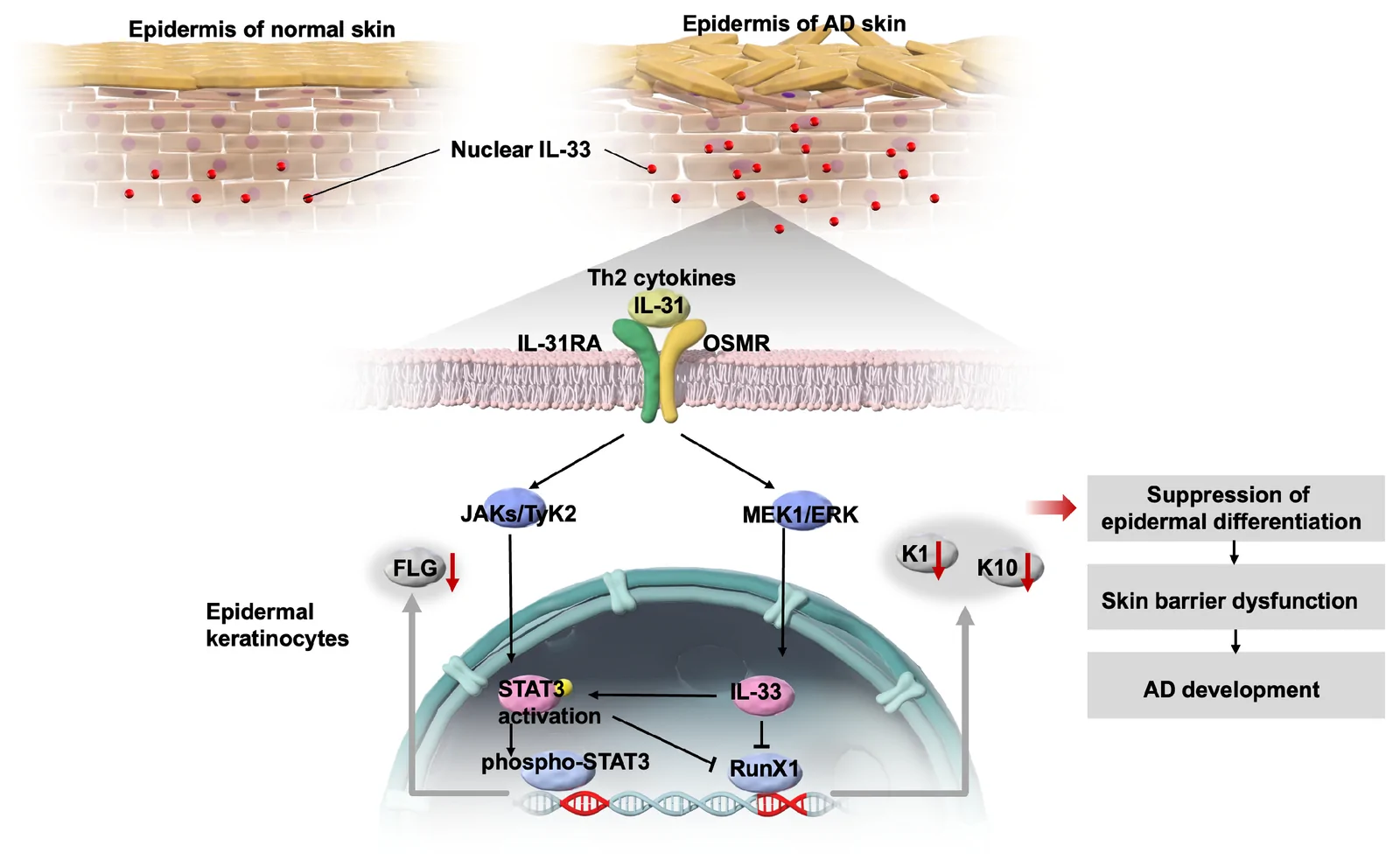
Nuclear IL-33 regulates STAT3 activation in IL-31-induced suppression of filaggrin and keratin 1/10 in keratinocytes. Adapted with permission from Ref. [11]. Copyright [2021], Elsevier.

Mainly produced by Th2 cells, IL-31 is elevated in the blood and skin of patients with AD and prurigo nodularis and contributes to chronic itch and barrier impairment [36,37]. We found that IL-31 stimulation increased nuclear IL-33 in keratinocytes. IL-31 activated STAT3 through its receptor, and nuclear IL-33 physically associated with pSTAT3 to form a complex, which enhanced STAT3 occupancy at the FLG promoter and suppressed FLG; moreover, the IL-33/pSTAT3 complex inhibited RUNX1 and reduced KRT1/KRT10 [11]. Therefore, IL-31 induces keratinocyte barrier impairment through a mechanism that is dependent on nuclear IL-33 (Figure 2). Considering that this barrier-disrupting effect depends on nuclear IL-33, it is mechanistically separable from the cytokine’s well-established, neuron-mediated pruritogenic action; this positions nuclear IL-33 as the intracellular hub that links chronic itch with concomitant epidermal barrier impairment.

### 6.3. TSLP

**Figure 3:**
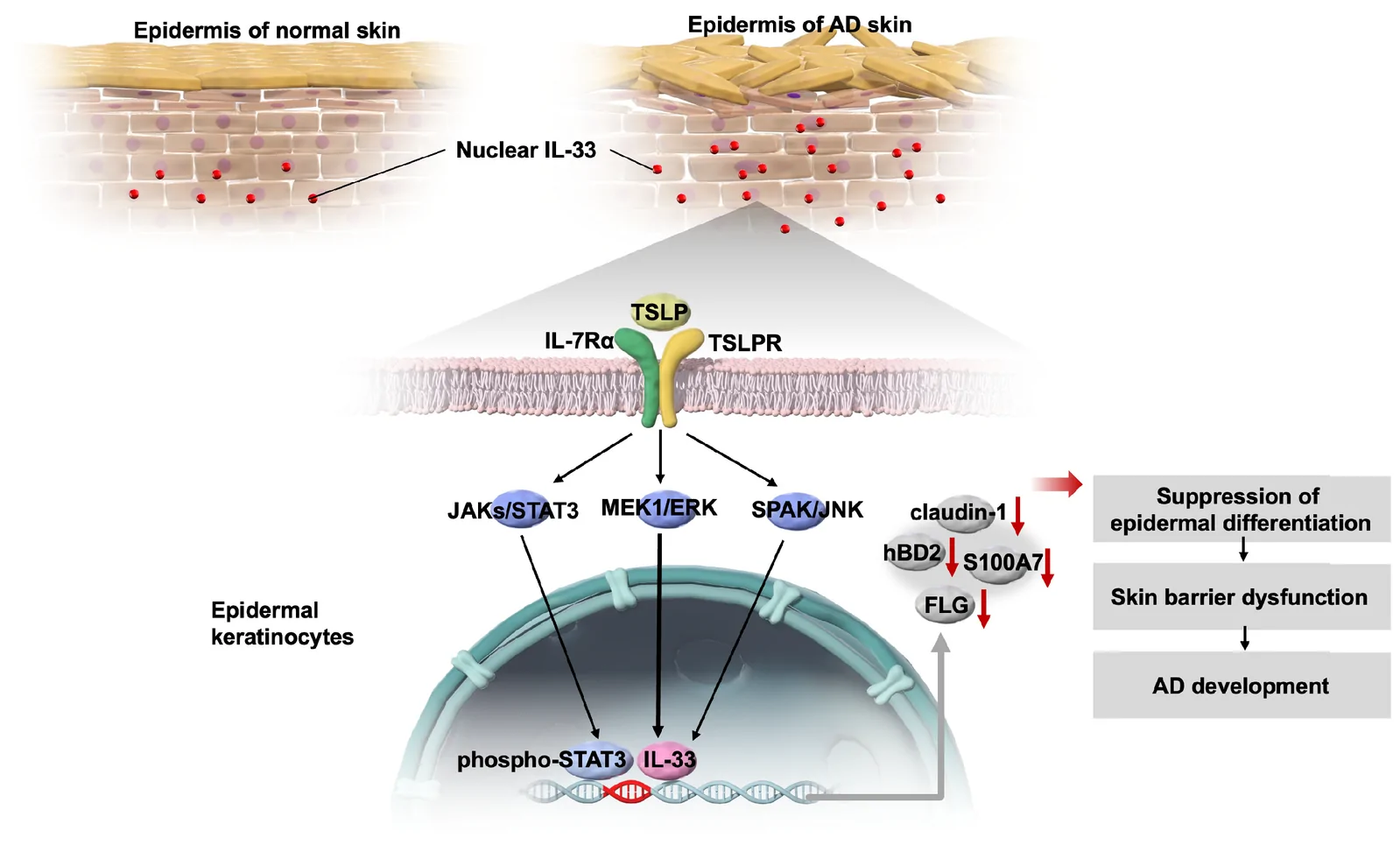
TSLP promotes the formation of a nuclear IL-33/phosphorylated STAT3 complex, which impairs the skin barrier in keratinocytes. Adapted with permission from Ref. [10]. Copyright [2022], Elsevier.

TSLP is a keratinocyte-derived cytokine that activates dendritic cells and Th2 cells and plays an important role in epidermal barrier function impairment during the early phase of AD [38,39]. Using human keratinocytes and three-dimensional skin models, we showed that nuclear IL-33 mediates TSLP-induced downregulation of FLG, the antimicrobial peptides hBD2 and S100A7, and the tight junction protein CLDN1. TSLP was reported to increase IL-33 expression and promote the formation of a nuclear IL-33/pSTAT3 complex, which bound the FLG promoter to repress filaggrin, and contributed to repression of hBD2, S100A7, and CLDN1 [10]. These findings suggested that TSLP impairs skin barrier function through nuclear IL-33, affecting both epidermal structural integrity and innate immune defense (Figure 3). By concurrently repressing structural proteins, tight junction components, and antimicrobial peptides, the nuclear IL-33/pSTAT3 complex compromises the coordinated physical, chemical, and immunological arms of epidermal defense. This offers a unifying explanation for the multidimensional barrier failure characteristic of AD.

### 6.4. Nuclear IL-33 as a Molecular Nexus Linking Epidermal Barrier Dysfunction and Innate Immune Dysregulation

Taken together, although mediated through their respective receptors, three distinct type 2 cytokine pathways (i.e., IL-4/IL-13, IL-31, and TSLP) converge on nuclear IL-33, which, in turn, appears to engage STAT3- and STAT6-dependent mechanisms to suppress a coordinated set of genes involved in epidermal barrier formation and keratinocyte differentiation. The prominent accumulation of nuclear IL-33 in lesional keratinocytes, as demonstrated by immunohistochemistry, was consistent with this model [12]. Functionally, the consequent reduction in FLG, LOR, CLDN1, and antimicrobial peptides may compromise both the physical and chemical components of the epidermal barrier, thereby facilitating allergen penetration. In parallel, scratching and barrier disruption may further promote alarmin release and amplify type 2 inflammation, thereby sustaining the characteristic vicious self-perpetuating inflammatory cycle of AD. From this perspective, elucidating the mechanisms that regulate nuclear IL-33 can be critical to achieving sustained disease control. Conceptually, this positions nuclear IL-33 as a shared convergence node rather than a downstream effector of any single cytokine; this property may help explain the clinical practice observation that interventions directed at individual upstream receptors achieved only partial barrier restoration.

### 6.5. Reconciling the Sequestration and Active-Regulator Paradigms

Our findings on keratinocytes may initially appear irreconcilable with the substantial evidence of endogenous nuclear IL-33 being transcriptionally inert in endothelial and certain epithelial cells [21,22]. Several considerations clarify this apparent contradiction. First, the barrier-suppressing activity of nuclear IL-33 in keratinocytes was observed specifically under a type 2 cytokine milieu, which drives STAT3 and STAT6 activation [11,12]; rather than acting as a classical constitutively active transcription factor, nuclear IL-33 may operate as a context-dependent transcriptional cofactor that requires an inflammatory signaling partner to manifest its effect. Such conditional activity would not be detected in resting cells. Second, the effects we observed were gene-specific, focused on *FLG* and *RUNX1* and their targets [12]; these could be easily missed by proteome- or transcriptome-wide screening, which emphasize global change. Third, differences in chromatin landscape and available partners among cell types likely determine whether the nuclear pool remains a silent reservoir or becomes a functional regulator. Therefore, we propose that nuclear sequestration and active intracellular regulation are not mutually exclusive but represent context- and cell type-dependent states of the same molecule; in the inflamed epidermis, the normally restrained nuclear pool is repurposed as a signaling hub of barrier dysfunction. Framed in this way, the sequestration and active regulator models described two ends of a continuum rather than a strict dichotomy, and the position along that continuum is determined by cell type, chromatin context, and the prevailing cytokine milieu. Importantly, because direct binding of IL-33 at the FLG or RUNX1 loci and a causal requirement for its nuclear localization have not yet been demonstrated, nuclear IL-33 is best regarded as a putative, context-dependent transcriptional cofactor rather than an established transcription factor, with a role likely to vary according to keratinocyte differentiation state, chromatin context, and the temporal stage of inflammation.

## 7. Therapeutic Implications

### 7.1. Targeting the Extracellular IL-33/ST2 Axis: Promise and Pitfalls

Given the compelling genetic and functional evidence linking the IL-33/ST2 axis with AD, IL-33 and ST2 emerged as attractive therapeutic targets, and early data initially seemed to support this concept. In a phase IIa study on 12 adults with moderate-to-severe AD, a single dose of the anti-IL-33 antibody etokimab produced rapid clinical benefits, with 83% of patients achieving a 50% reduction in the Eczema Area and Severity Index and a decrease in peripheral eosinophil levels [40]. However, a subsequent larger phase IIb study on approximately 300 patients failed to show a significant advantage over a placebo at week 16 [41]. Two phase II trials on the anti-IL-33 antibody itepekimab, including a combination arm with dupilumab, were terminated early after failing to improve AD outcomes; likewise, the anti-IL-33 antibody LY3375880 was found to be ineffective in AD [42]. In another phase II AD trial, the anti-ST2 antibody astegolimab failed to show superiority over a placebo, in part due to a high placebo response [41]. Other agents targeting this axis include the anti-IL-33 antibody PF-06817024 [43] and the anti-ST2 antibody melrilimab (CNTO7160/GSK3772847) [44], while the trispecific antibody ompekimig (PF-07264660; ClinicalTrials.gov NCT05995964) blocks IL-4, IL-13, and IL-33 simultaneously. Beyond antibodies, small-molecule ST2 inhibitors [45] and natural compounds such as resveratrol [46], quercetin [47], and tranilast [48]—and, with more limited evidence, honokiol—have shown preclinical modulation of the IL-33/ST2 pathway (Supplementary Table S1).

Notably, targeting the same axis has shown clearer signals in airway disease. In particular, astegolimab significantly reduced exacerbations in severe asthma [49] and, compared with a placebo, improved health status in COPD, although it failed to reduce exacerbations [50]. The contrast between airway and skin outcomes suggested that (1) the contribution of IL-33 is context- and endotype-dependent; (2) redundancy among type 2 cytokines limits the effect of neutralizing IL-33 alone; and, as we would argue, (3) extracellular neutralization cannot reach the intracellular nuclear pool of IL-33, which may repress barrier genes in keratinocytes. Recent structural studies further refined the concept that differential epitope recognition among clinical anti-IL-33 antibodies, which could yield either complete or partial disruption of ST2 binding, provides a molecular rationale for divergent efficacy [51]. These observations provided a rationale for strategies that act downstream or upstream of the released cytokine. Critically, however, all these agents act solely on extracellular IL-33, and none engage the nuclear compartment, which may preserve the pathogenic ST2-independent activity in keratinocytes. Given that extracellular blockade fails to neutralize the nuclear pool, which is implicated in ST2-independent transcriptional regulation of the epidermal barrier, these observations offer one hypothesis-generating rationale for complementary strategies aimed at the nuclear pool; however, the limited efficacy of IL-33/ST2 blockade in AD is more likely multifactorial—reflecting cytokine redundancy, endotype heterogeneity, pharmacodynamic factors, and trial design (including high placebo responses)—rather than due to any single mechanism.

On the other hand, dupilumab, which blocks the shared IL-4 receptor alpha chain and neutralizes IL-4 and IL-13 signaling, has transformed the management of moderate-to-severe AD. This indicates that simultaneous interruption of multiple type 2 signals upstream of the barrier may be more effective than neutralizing a single alarmin. This clinical experience is consistent with the view that IL-33 operates within a redundant cytokine network and that its most consequential action in keratinocytes may be intracellular rather than due to secretion.

Within this broader therapeutic landscape, the IL-13-selective antibodies tralokinumab and lebrikizumab have also proven effective in moderate-to-severe AD [52,53], reinforcing the central role of the IL-4/IL-13 effector axis, whereas topical calcineurin inhibitors such as tacrolimus act more proximally by blocking NFAT-driven transcription of type 2 cytokines in T cells [54]. Against these broadly acting, established therapies, IL-33- and ST2-directed strategies can be organized by their principal target-extracellular IL-33 (e.g., etokimab, itepekimab), the ST2/IL1RL1 receptor (e.g., astegolimab), the upstream keratinocyte-derived and nuclear IL-33 pool (e.g., topical trehalose), and combined IL-33/type 2 blockade—each engaging a distinct point along the alarmin-to-effector cascade (Supplementary Table S1).

### 7.2. Downstream Targeting: JAK/STAT Inhibition

Because nuclear IL-33 exerts its barrier-suppressing effect through STAT3 and STAT6, inhibitors of the JAK/STAT pathway represent a logical downstream strategy. The topical JAK inhibitor delgocitinib has been reported to improve skin barrier function by suppressing STAT3 signaling [55]. Viewed through the mechanism of the nuclear IL-33 hub, part of the barrier restoration benefit of JAK inhibition may reflect interruption of the nuclear IL-33/STAT3 axis, offering a mechanistic reinterpretation of an existing therapeutic class. Consistent with this interpretation, orally administered JAK inhibitors, such as abrocitinib and upadacitinib, were reported to produce rapid and substantial clinical improvement in AD; this benefit may be partly due to attenuation of STAT3- and STAT6-dependent barrier repression downstream of the nuclear IL-33 hub.

### 7.3. Nuclear IL-33 as a Therapeutic Target

In principle, selective control of the nuclear IL-33 signal could simultaneously restore barrier function and dampen inflammation. Given the current unavailability of a direct pharmacological nuclear IL-33 inhibitor for clinical use, we explored alternative approaches. Trehalose is a nonreducing disaccharide with well-established cytoprotective activity against desiccation stress. We previously found that high-concentration trehalose can transiently convert human fibroblasts into a prohealing phenotype [56]. Therefore, we tested its effect on keratinocyte barrier impairment induced by type 2 cytokine [9]. This rationale motivated a search for agents that are capable of decreasing nuclear IL-33 burden through indirect upstream mechanisms rather than by direct molecular inhibition.

**Figure 4:**
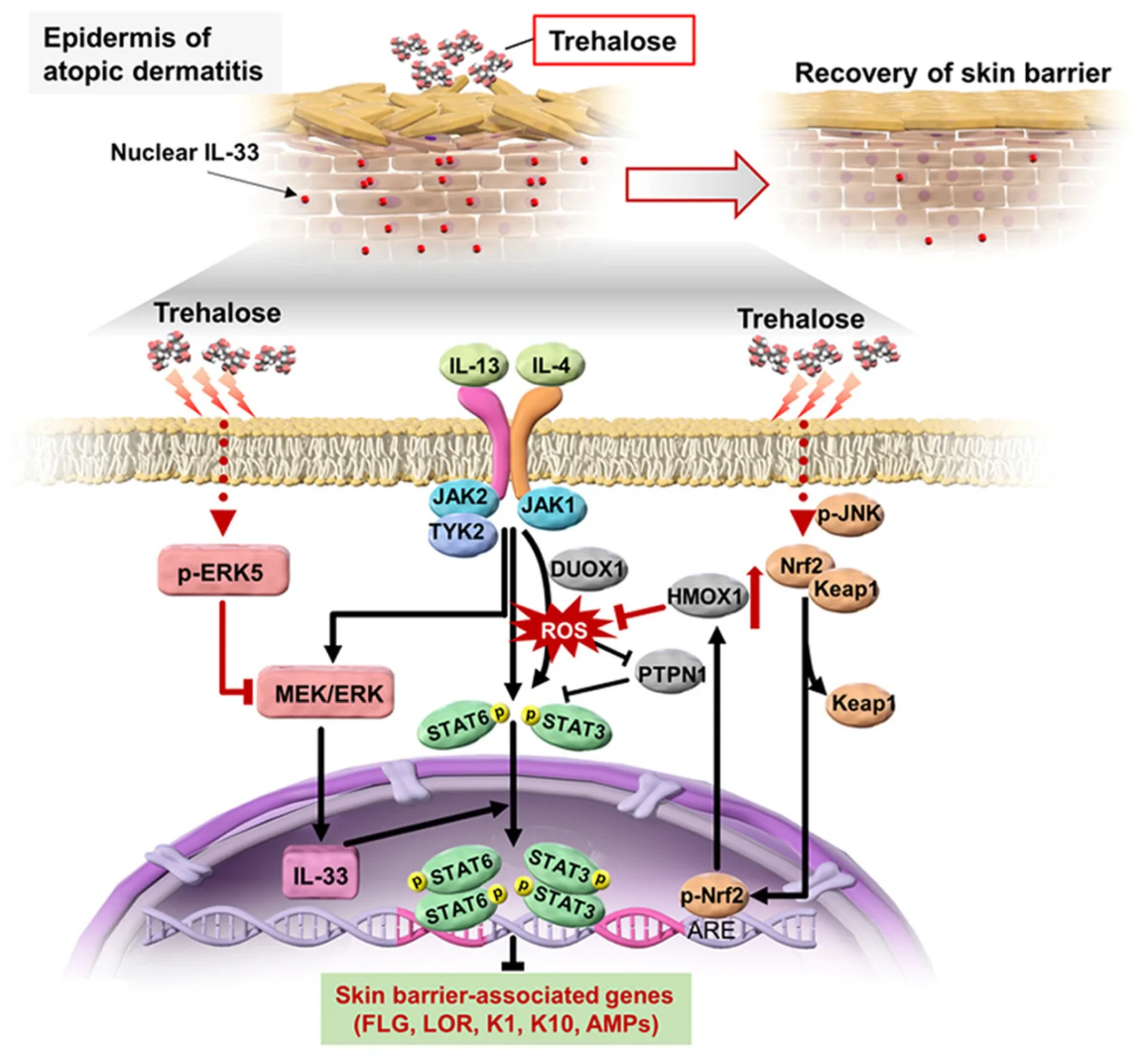
Trehalose repairs IL-4/IL-13-induced keratinocyte barrier impairment through suppression of nuclear IL-33 and activation of antioxidant (NRF2) responses. Adapted with permission from Ref. [9]. Copyright [2025], Elsevier.

Comprehensive RNA sequencing showed that trehalose treatment restored the expression of numerous barrier-related genes in human keratinocytes. In an IL-4/IL-13-stimulated model of AD-like barrier impairment, both monolayer keratinocytes and three-dimensional skin equivalents showed reduced FLG, LOR, KRT1/KRT10, and hBD2, together with impaired barrier function; all of these were significantly recovered by high-concentration trehalose. Mechanistically, suppression of IL-33 expression and a marked reduction in nuclear IL-33 protein by trehalose were associated with ERK1/2 pathway inhibition and ERK5 pathway activation; therefore, trehalose precisely counteracts IL-4/IL-13-induced increases in nuclear IL-33 via ERK1/2 [9,12]. Moreover, trehalose was shown to activate the antioxidant master regulator NRF2; by stimulating the JNK pathway, it promoted nuclear translocation of NRF2 and induction of antioxidant enzymes, thereby mitigating the oxidative stress generated by IL-4/IL-13. Through these combined actions, trehalose attenuated excessive activation of STAT3 and STAT6 and repaired barrier function (Figure 4) [9].

Although based on in vitro systems, these findings introduced the concept of molecularly targeted skincare, which is mechanistically different from conventional emollients. Trehalose is a comparatively safe sugar that is already being used in food products and cosmetics [57], although controlled clinical studies of trehalose-containing topical formulations remain warranted. Nevertheless, more broadly, this work illustrated the feasibility of targeting the nuclear IL-33 pathway topically and supported a new paradigm in barrier-directed AD therapy. Given that trehalose acts intracellularly to reduce nuclear IL-33 burden while simultaneously reinforcing antioxidant defenses, it exemplifies a mechanistic class that is distinct from cytokine-neutralizing biologics and broadly acting immunosuppressants. Realizing this potential will require translating an in vitro mechanism into a clinically validated topical therapy through rigorous vehicle-controlled evaluation, and sustainability of barrier benefits under real-world conditions is yet to be established. Should these conditions be met, nuclear IL-33-directed topical agents may open a new therapeutic avenue toward durable barrier restoration in AD.

### 7.4. Counter-Regulation and Future Targets

The balance of the IL-33 axis is shaped by counterregulatory molecules. IL-37, which is an anti-inflammatory member of the IL-1 family, can act antagonistically to IL-33. Dissecting the interaction of such regulators alongside nuclear IL-33 may enable comprehensive strategies that can simultaneously maintain barrier integrity and inhibit inflammation. A more complete understanding of the IL-33 system endogenous brakes, which include the decoy receptor sST2, anti-inflammatory cytokine IL-37, and oxidative inactivation of the released alarmin, may point toward therapies that reinforce the skin’s own regulatory mechanisms rather than relying on exogenous cytokine blockade, thereby restoring the physiological restraint on the IL-33 system, which fails in AD, wherein pathological accumulation of nuclear IL-33 suppresses epidermal barrier function.

## 8. Open Questions, Limitations, and Future Directions

Several gaps must be addressed before the nuclear IL-33 model can be fully translated. Most evidence for its active nuclear function in keratinocytes is based on in vitro monolayer and three-dimensional culture systems. Keratinocyte-specific, nuclear domain-targeted in vivo models, such as animals carrying CBM/NLS mutations restricted to the epidermis, are needed to establish causality in intact skin. At the mechanistic level, the function of nuclear IL-33 as a transcriptional cofactor for STAT3 remains to be proven, perhaps through direct measurement of chromatin occupancy at the *FLG* and *RUNX1* loci and structural characterization of the nuclear IL-33/STAT3 complex. Reconciling keratinocyte data with the apparently inert nuclear pool in endothelial and some epithelial cells will require comparative studies among different cell types. Single-cell and spatial transcriptomic approaches to lesional and nonlesional skin could help define the keratinocyte subpopulations that harbor transcriptionally active nuclear IL-33 and describe the evolution of this state across the transition of AD from acute to chronic. The current levels of evidence underlying the nuclear IL-33 model are summarized in Supplementary Table S2.

Clinically, the heterogeneous results of IL-33/ST2-targeting trials highlighted the need for biomarkers, such as lesional nuclear IL-33 abundance or circulating sST2, to identify the AD endotypes most likely to respond and to explain the divergence between skin and airway outcomes. Translational priorities include well-designed trials on topical trehalose and other nuclear IL-33-directed approaches, including their safety, efficacy, and rational combination with emollients or anti-inflammatory agents. Finally, because nuclear IL-33 is expressed throughout barrier epithelia, its role in other barrier-associated diseases, such as asthma, inflammatory bowel disease, and psoriasis, deserves comparative investigation. Ultimately, integration of molecular endotyping with barrier- and immune-directed interventions will be essential to improve the precision of mechanism-based management of AD.

## 9. Conclusions

IL-33 is far more than a Th2-promoting alarmin. In the inflamed epidermis, the abundant nuclear pool of IL-33 may act as an intracellular hub that links signals from IL-4/IL-13, IL-31, and TSLP to STAT3- and STAT6-mediated inhibition of FLG, LOR, keratins, CLDN1, and antimicrobial peptides, placing nuclear IL-33 at the center of the vicious barrier–inflammation cycle that sustains AD. This view reframes nuclear IL-33 as a context-dependent epidermal barrier regulator rather than a passively stored cytokine and helps explain the insufficient effects of neutralizing the extracellular cytokine alone in AD. In vitro demonstration that agents such as trehalose can safely suppress nuclear IL-33 and restore barrier function provided proof of concept for molecularly targeted, barrier-restorative topical therapy. Continued basic and clinical investigation of the nuclear IL-33 pathway should advance the understanding of AD pathophysiology and development of treatments aimed at durable remission. More broadly, recognition of the fact that a nuclear cytokine can be repurposed as an intracellular transcriptional regulator under inflammatory conditions expands the conceptual landscape of alarmin biology and invites reexamination of nuclear cytokine functions across the spectrum of barrier-associated inflammatory diseases. These conclusions derive largely from in vitro keratinocyte and three-dimensional skin models; establishing the causal, context-dependent role of nuclear IL-33 in vivo and in lesional human skin remains an essential next step.

## Data Availability

No new data were created or analyzed in this study. Data sharing is not applicable to this article.

## Supplementary Materials

The following supporting information can be downloaded at: https://www.mdpi.com/article/10.3390/jcm15207781/s1, Table S1. Therapeutic strategies and agents targeting the IL-33 axis in atopic dermatitis and allied type 2 diseases. Table S2. Levels of evidence for the nuclear IL-33 model of type 2 cytokine-induced epidermal barrier dysfunction.

## Abbreviations

The following abbreviations are used in this manuscript:

AD	atopic dermatitis
CBM	chromatin-binding motif
CLDN1	claudin-1
DAMP	damage-associated molecular pattern
EASI	Eczema Area and Severity Index
ERK	extracellular signal-regulated kinase
FLG	filaggrin
hBD2	human beta-defensin 2
IL	interleukin
IL-1RAcP	IL-1 receptor accessory protein
ILC2	group 2 innate lymphoid cell
IRAK	interleukin-1 receptor-associated kinase
JNK	c-Jun N-terminal kinase
KRT	keratin
LOR	loricrin
MAPK	mitogen-activated protein kinase
MEK	MAPK/ERK kinase
MyD88	myeloid differentiation primary response protein 88
NF-κB	nuclear factor kappa-light-chain-enhancer of activated B cells
NK cell	natural killer cell
NLS	nuclear localization sequence
NRF2	nuclear factor erythroid 2-related factor 2
PN	prurigo nodularis
pSTAT3	phosphorylated signal transducer and activator of transcription 3
RUNX1	runt-related transcription factor 1
sST2	soluble ST2
STAT	signal transducer and activator of transcription
ST2	suppression of tumorigenicity 2 (IL1RL1, interleukin-1 receptor-like 1)
TEWL	transepidermal water loss
Th cell	T helper cell
Treg	regulatory T cell
TRAF6	TNF receptor-associated factor 6
TSLP	thymic stromal lymphopoietin

## References

[1] Schmitz J., Owyang A., Oldham E., Song Y., Murphy E., McClanahan T.K., Zurawski G., Moshrefi M., Qin J., Li X. (2005). IL-33, an interleukin-1-like cytokine that signals via the IL-1 receptor-related protein ST2 and induces T helper type 2-associated cytokines. Immunity.

[2] Cayrol C., Girard J.P. (2018). Interleukin-33 (IL-33): A nuclear cytokine from the IL-1 family. Immunol. Rev..

[3] Liew F.Y., Girard J.P., Turnquist H.R. (2016). Interleukin-33 in health and disease. Nat. Rev. Immunol..

[4] Moussion C., Ortega N., Girard J.-P. (2008). The IL-1-Like Cytokine IL-33 Is Constitutively Expressed in the Nucleus of Endothelial Cells and Epithelial Cells In Vivo: A Novel ‘Alarmin’?. PLoS ONE.

[5] Savinko T., Matikainen S., Saarialho-Kere U., Lehto M., Wang G., Lehtimäki S., Karisola P., Reunala T., Wolff H., Lauerma A. (2012). IL-33 and ST2 in Atopic Dermatitis: Expression Profiles and Modulation by Triggering Factors. J. Investig. Dermatol..

[6] Imai Y., Yasuda K., Sakaguchi Y., Haneda T., Mizutani H., Yoshimoto T., Nakanishi K., Yamanishi K. (2013). Skin-specific expression of IL-33 activates group 2 innate lymphoid cells and elicits atopic dermatitis-like inflammation in mice. Proc. Natl. Acad. Sci. USA.

[7] Carriere V., Roussel L., Ortega N., Lacorre D.-A., Americh L., Aguilar L., Bouche G., Girard J.-P. (2007). IL-33, the IL-1-like cytokine ligand for ST2 receptor, is a chromatin-associated nuclear factor in vivo. Proc. Natl. Acad. Sci. USA.

[8] Roussel L., Erard M., Cayrol C., Girard J.P. (2008). Molecular mimicry between IL-33 and KSHV for attachment to chromatin through the H2A-H2B acidic pocket. EMBO Rep..

[9] Dai X., Mizukami Y., Watanabe K., Tsuda T., Shidahara M., Yoshida S., Yatsuzuka K., Shiraishi K., Mori H., Murakami M. (2025). Trehalose Prevents IL-4/IL-13-Induced Skin Barrier Impairment by Suppressing IL-33 Expression and Increasing NRF2 Activation in Human Keratinocytes In Vitro. J. Investig. Dermatol..

[10] Dai X., Muto J., Shiraishi K., Utsunomiya R., Mori H., Murakami M., Sayama K. (2022). TSLP Impairs Epidermal Barrier Integrity by Stimulating the Formation of Nuclear IL-33/Phosphorylated STAT3 Complex in Human Keratinocytes. J. Investig. Dermatol..

[11] Dai X., Shiraishi K., Muto J., Utsunomiya R., Mori H., Murakami M., Sayama K. (2021). Nuclear IL-33 plays an important role in IL-31-mediated downregulation of filaggrin, keratin 1, and keratin 10 by regulating STAT3 activation in human keratinocytes. J. Investig. Dermatol..

[12] Dai X., Utsunomiya R., Shiraishi K., Mori H., Muto J., Murakami M., Sayama K. (2021). Nuclear IL-33 Plays an Important Role in the Suppression of FLG, LOR, Keratin 1, and Keratin 10 by IL-4 and IL-13 in Human Keratinocytes. J. Investig. Dermatol..

[13] Yasuda K., Muto T., Kawagoe T., Matsumoto M., Sasaki Y., Matsushita K., Taki Y., Futatsugi-Yumikura S., Tsutsui H., Ishii K.J. (2012). Contribution of IL-33-activated type II innate lymphoid cells to pulmonary eosinophilia in intestinal nematode-infected mice. Proc. Natl. Acad. Sci. USA.

[14] Barlow J.L., Peel S., Fox J., Panova V., Hardman C.S., Camelo A., Bucks C., Wu X., Kane C.M., Neill D.R. (2013). IL-33 is more potent than IL-25 in provoking IL-13-producing nuocytes (type 2 innate lymphoid cells) and airway contraction. J. Allergy Clin. Immunol..

[15] Nakanishi W., Yamaguchi S., Matsuda A., Suzukawa M., Shibui A., Nambu A., Kondo K., Suto H., Saito H., Matsumoto K. (2013). IL-33, but not IL-25, is crucial for the development of house dust mite antigen-induced allergic rhinitis. PLoS ONE.

[16] Bessa J., Meyer C.A., de Vera Mudry M.C., Schlicht S., Smith S.H., Iglesias A., Cote-Sierra J. (2014). Altered subcellular localization of IL-33 leads to non-resolving lethal inflammation. J. Autoimmun..

[17] Travers J., Rochman M., Miracle C.E., Habel J.E., Brusilovsky M., Caldwell J.M., Rymer J.K., Rothenberg M.E. (2018). Chromatin regulates IL-33 release and extracellular cytokine activity. Nat. Commun..

[18] Lefrancais E., Roga S., Gautier V., Gonzalez-de-Peredo A., Monsarrat B., Girard J.P., Cayrol C. (2012). IL-33 is processed into mature bioactive forms by neutrophil elastase and cathepsin G. Proc. Natl. Acad. Sci. USA.

[19] Luthi A.U., Cullen S.P., McNeela E.A., Duriez P.J., Afonina I.S., Sheridan C., Brumatti G., Taylor R.C., Kersse K., Vandenabeele P. (2009). Suppression of interleukin-33 bioactivity through proteolysis by apoptotic caspases. Immunity.

[20] Cohen E.S., Scott I.C., Majithiya J.B., Rapley L., Kemp B.P., England E., Rees D.G., Overed-Sayer C.L., Woods J., Bond N.J. (2015). Oxidation of the alarmin IL-33 regulates ST2-dependent inflammation. Nat. Commun..

[21] Gautier V., Cayrol C., Farache D., Roga S., Monsarrat B., Burlet-Schiltz O., Gonzalez de Peredo A., Girard J.P. (2016). Extracellular IL-33 cytokine, but not endogenous nuclear IL-33, regulates protein expression in endothelial cells. Sci. Rep..

[22] He Z., Chen L., Furtado G.C., Lira S.A. (2018). Interleukin 33 regulates gene expression in intestinal epithelial cells independently of its nuclear localization. Cytokine.

[23] Gatti F., Mia S., Hammarström C., Frerker N., Fosby B., Wang J., Pietka W., Sundnes O., Hol J., Kasprzycka M. (2021). Nuclear IL-33 restrains the early conversion of fibroblasts to an extracellular matrix-secreting phenotype. Sci. Rep..

[24] Ali S., Mohs A., Thomas M., Klare J., Ross R., Schmitz M.L., Martin M.U. (2011). The dual function cytokine IL-33 interacts with the transcription factor NF-kappaB to dampen NF-kappaB-stimulated gene transcription. J. Immunol..

[25] Guttman-Yassky E., Renert-Yuval Y., Brunner P.M. (2025). Atopic dermatitis. Lancet.

[26] Imai Y. (2019). Interleukin-33 in atopic dermatitis. J. Dermatol. Sci..

[27] Seltmann J., Roesner L.M., von Hesler F.W., Wittmann M., Werfel T. (2015). IL-33 impacts on the skin barrier by downregulating the expression of filaggrin. J. Allergy Clin. Immunol..

[28] Ryu W.-I., Lee H., Bae H.C., Ryu H.J., Son S.W. (2016). IL-33 down-regulates filaggrin expression by inducing STAT3 and ERK phosphorylation in human keratinocytes. J. Dermatol. Sci..

[29] Ryu W.-I., Lee H., Bae H.C., Jeon J., Ryu H.J., Kim J., Kim J.H., Son J.W., Kim J., Imai Y. (2018). IL-33 down-regulates CLDN1 expression through the ERK/STAT3 pathway in keratinocytes. J. Dermatol. Sci..

[30] Berdyshev E., Goleva E., Bronova I., Dyjack N., Rios C., Jung J., Taylor P., Jeong M., Hall C.F., Richers B.N. (2018). Lipid abnormalities in atopic skin are driven by type 2 cytokines. JCI Insight.

[31] Trier A.M., Mack M.R., Fredman A., Tamari M., Ver Heul A.M., Zhao Y., Guo C.J., Avraham O., Ford Z.K., Oetjen L.K. (2022). IL-33 signaling in sensory neurons promotes dry skin itch. J. Allergy Clin. Immunol..

[32] Liu B., Tai Y., Achanta S., Kaelberer M.M., Caceres A.I., Shao X., Fang J., Jordt S.E. (2016). IL-33/ST2 signaling excites sensory neurons and mediates itch response in a mouse model of poison ivy contact allergy. Proc. Natl. Acad. Sci. USA.

[33] Theoharides T.C., Zhang B., Kempuraj D., Tagen M., Vasiadi M., Angelidou A., Alysandratos K.D., Kalogeromitros D., Asadi S., Stavrianeas N. (2010). IL-33 augments substance P-induced VEGF secretion from human mast cells and is increased in psoriatic skin. Proc. Natl. Acad. Sci. USA.

[34] Zhao J., Zhou L., Wang C., Rao Z., Yuan X., Cui J., Zhao D., Sun Y., Chen Y., Li R. (2026). Epidermal PAR2-TRPV3-IL-33 Signaling Promotes Mast Cell Recruitment and Sensory Nerve-Mast Cell Interactions in Atopic Dermatitis. Allergy.

[35] Steinhoff M., Ahmad F., Pandey A., Datsi A., AlHammadi A., Al-Khawaga S., Al-Malki A., Meng J., Alam M., Buddenkotte J. (2022). Neuroimmune communication regulating pruritus in atopic dermatitis. J. Allergy Clin. Immunol..

[36] Yokozeki H., Murota H., Matsumura T., Komazaki H., Nemolizumab-JP11 Study Group (2024). Efficacy and safety of nemolizumab and topical corticosteroids for prurigo nodularis: Results from a randomized double-blind placebo-controlled phase II/III clinical study in patients aged ≥ 13 years. Br. J. Dermatol..

[37] Kabashima K., Matsumura T., Komazaki H., Kawashima M., Nemolizumab J.P.a.S.G. (2022). Nemolizumab plus topical agents in patients with atopic dermatitis (AD) and moderate-to-severe pruritus provide improvement in pruritus and signs of AD for up to 68 weeks: Results from two phase III, long-term studies. Br. J. Dermatol..

[38] Soumelis V., Reche P.A., Kanzler H., Yuan W., Edward G., Homey B., Gilliet M., Ho S., Antonenko S., Lauerma A. (2002). Human epithelial cells trigger dendritic cell mediated allergic inflammation by producing TSLP. Nat. Immunol..

[39] Wang S.H., Zuo Y.G. (2021). Thymic Stromal Lymphopoietin in Cutaneous Immune-Mediated Diseases. Front. Immunol..

[40] Chen Y.L., Gutowska-Owsiak D., Hardman C.S., Westmoreland M., MacKenzie T., Cifuentes L., Waithe D., Lloyd-Lavery A., Marquette A., Londei M. (2019). Proof-of-concept clinical trial of etokimab shows a key role for IL-33 in atopic dermatitis pathogenesis. Sci. Transl. Med..

[41] Maurer M., Cheung D.S., Theess W., Yang X., Dolton M., Guttman A., Choy D.F., Dash A., Grimbaldeston M.A., Soong W. (2022). Phase 2 randomized clinical trial of astegolimab in patients with moderate to severe atopic dermatitis. J. Allergy Clin. Immunol..

[42] Kosloski M.P., Guttman-Yassky E., Cork M.J., Worm M., Nahm D.H., Zhu X., Ruddy M.K., Harel S., Kamal M.A., Goulaouic H. (2024). Pharmacokinetics and pharmacodynamics of itepekimab in adults with moderate-to-severe atopic dermatitis: Results from two terminated phase II trials. Clin. Transl. Sci..

[43] Danto S.I., Tsamandouras N., Reddy P., Gilbert S., Mancuso J., Page K., Peeva E., Vincent M.S., Beebe J.S. (2024). Safety, Tolerability, Pharmacokinetics, and Pharmacodynamics of PF-06817024 in Healthy Participants, Participants with Chronic Rhinosinusitis with Nasal Polyps, and Participants with Atopic Dermatitis: A Phase 1, Randomized, Double-Blind, Placebo-Controlled Study. J. Clin. Pharmacol..

[44] Nnane I., Frederick B., Yao Z., Raible D., Shu C., Badorrek P., van den Boer M., Branigan P., Duffy K., Baribaud F. (2020). The first-in-human study of CNTO 7160, an anti-interleukin-33 receptor monoclonal antibody, in healthy subjects and patients with asthma or atopic dermatitis. Br. J. Clin. Pharmacol..

[45] Yuan X., Jiang H., Fu D., Robida A., Rajanayake K., Yuan H., Wen B., Sun D., Watch B.T., Chinnaswamy K. (2022). Structure-Activity relationship of 1-(Furan-2ylmethyl)Pyrrolidine-Based Stimulation-2 (ST2) inhibitors for treating graft versus host disease. Bioorganic Med. Chem..

[46] Nakajima S., Ishimaru K., Kobayashi A., Yu G., Nakamura Y., Oh-Oka K., Suzuki-Inoue K., Kono K., Nakao A. (2019). Resveratrol inhibits IL-33-mediated mast cell activation by targeting the MK2/3-PI3K/Akt axis. Sci. Rep..

[47] Xiao Y., Xu M., Zhong L., Lin F., Yu M., Luo H., Lin C. (2026). Quercetin modulates mast cell activation and the IL-33/ST2/NF-kappaB axis in allergic rhinitis. Sci. Rep..

[48] Tsuji K., Fukuda K., Fukushima A. (2017). Inhibition by Tranilast of the Synergistic Induction of Degranulation and IL-13 Expression by IL-33 and FcvarepsilonRI Cross-linking in Mast Cells. Ocul. Immunol. Inflamm..

[49] Kelsen S.G., Agache I.O., Soong W., Israel E., Chupp G.L., Cheung D.S., Theess W., Yang X., Staton T.L., Choy D.F. (2021). Astegolimab (anti-ST2) efficacy and safety in adults with severe asthma: A randomized clinical trial. J. Allergy Clin. Immunol..

[50] Yousuf A.J., Mohammed S., Carr L., Yavari Ramsheh M., Micieli C., Mistry V., Haldar K., Wright A., Novotny P., Parker S. (2022). Astegolimab, an anti-ST2, in chronic obstructive pulmonary disease (COPD-ST2OP): A phase 2a, placebo-controlled trial. Lancet Respir. Med..

[51] Chen J., Wang Y., Wang X. (2026). Structures of clinical antibodies bound to IL-33 uncover two distinct epitopes underlying differential efficacy. MAbs.

[52] Wollenberg A., Blauvelt A., Guttman-Yassky E., Worm M., Lynde C., Lacour J.P., Spelman L., Katoh N., Saeki H., Poulin Y. (2021). Tralokinumab for moderate-to-severe atopic dermatitis: Results from two 52-week, randomized, double-blind, multicentre, placebo-controlled phase III trials (ECZTRA 1 and ECZTRA 2). Br. J. Dermatol..

[53] Silverberg J.I., Guttman-Yassky E., Thaci D., Irvine A.D., Stein Gold L., Blauvelt A., Simpson E.L., Chu C.Y., Liu Z., Gontijo Lima R. (2023). Two Phase 3 Trials of Lebrikizumab for Moderate-to-Severe Atopic Dermatitis. N. Engl. J. Med..

[54] Ruzicka T., Bieber T., Schopf E., Rubins A., Dobozy A., Bos J.D., Jablonska S., Ahmed I., Thestrup-Pedersen K., Daniel F. (1997). A short-term trial of tacrolimus ointment for atopic dermatitis. European Tacrolimus Multicenter Atopic Dermatitis Study Group. N. Engl. J. Med..

[55] Amano W., Nakajima S., Kunugi H., Numata Y., Kitoh A., Egawa G., Dainichi T., Honda T., Otsuka A., Kimoto Y. (2015). The Janus kinase inhibitor JTE-052 improves skin barrier function through suppressing signal transducer and activator of transcription 3 signaling. J. Allergy Clin. Immunol..

[56] Muto J., Fukuda S., Watanabe K., Dai X., Tsuda T., Kiyoi T., Kameda K., Kawakami R., Mori H., Shiraishi K. (2023). Highly concentrated trehalose induces prohealing senescence-like state in fibroblasts via CDKN1A/p21. Commun. Biol..

[57] Richards A.B., Krakowka S., Dexter L.B., Schmid H., Wolterbeek A.P., Waalkens-Berendsen D.H., Shigoyuki A., Kurimoto M. (2002). Trehalose: A review of properties, history of use and human tolerance, and results of multiple safety studies. Food Chem. Toxicol..

